# The diverse lives of TRAP1

**DOI:** 10.18632/oncoscience.84

**Published:** 2014-09-22

**Authors:** Jackeline Agorreta, Jiangting Hu, Francesco Pezzella

**Affiliations:** Oncology Division, Center for Applied Medical Research (CIMA), University of Navarra, Pamplona, Spain; Nuffield Department of Clinical Laboratory Sciences, University of Oxford, John Radcliffe Hospital, Oxford, United Kingdom

TNFR-associated protein1 (TRAP1) belongs to the heat shock protein 90 family of chaperons, and it is involved in protein quality control. Although TRAP1 can be localized in the cytosol and nucleus, it is mainly expressed in the mitochondria where it is involved in protection from apoptosis by different mechanisms. TRAP1 interacts with Cyclophillin D1, a protein which controls the function of the Permeability Transition Pore (PTP); in its absence, Cyclophillin D1 is activated, opening the PTP with consequent mitochondrial swelling and eventual apoptosis [1]. TRAP1 is also important in maintaining the cellular metabolic homeostasis mainly by preserving the tethering to the mitochondrial membrane of Hex II and by keeping active the Complex 2 of the respiratory chain. In its absence HexII is released inside the cytoplasm and is inactivated, leading to a proportional reduction of glycolysis and Oxidative Phosphorilation (OxPhos) of the respiratory chain. Furthermore Complex2 is inactivated with an extra impairment of OxPhos activity resulting in an added reduction of ATP production [2,3].

However TRAP1 is not only localized in the mitochondria: it has also been found associated with the intracellular domain of the type 1 receptor for the tumour necrosis factor (TNFR-1IC), on the endoplasmic reticulum, and in the nucleus. Consequently it is likely to be controlling a large number of pathways and not just in the mitochondria. TRAP1 was originally described as chaperon to Retinoblastoma (RB1) [4], a nuclear protein, and has the ability of maintain RB active. These results, together with our observations that in some tumours TRAP1 mRNA was actually lower than in the correspondent normal tissue [5], led us to raise the hypothesis that its loss could, in some circumstances, provide a growth advantage by causing misfolding and subsequent inactivation of the tumour suppressor protein RB1.

Therefore we performed a functional analysis of TRAP1, which focused on its influence on cell cycle regulation in relation to RB1. Following a chemical or physical shock, TRAP1 translocates to the nucleus and maintains RB1 in active conformation. When it is silenced by siRNA, or prevented from entering the nucleus in hypoxic cells (e.g. by geldanamycin), formation of RB1/E2F1 complexes is impaired because of misfolding of Rb1 protein which consequently fails to block cell cycle progression at the G1/S transition [5].

This action of TRAP1, due to his chaperoning function in an extra mitochondrial location, is however transient as, following the induction of hypoxia or heath shock, increased levels of nuclear TRAP1 are observed for a limited period between 16 and 24 hours. No effect is seen on normoxic cells.

In fact, when we explored the more longer term consequences of its silencing on cell growth in normoxic conditions [6], we found that TRAP1 knockdown reduces “in vitro” lung cancer cell growth and survival, reducing the number of cells entering in G2-M phase. Interestingly enough this happens in normoxic cells and therefore this effect, which start to appear instead between 24 and 48 hours in culture, is likely to be due to actions other than chaperoning as the cells are not subject to any shock requiring it. No differences due to silencing by siRNA were seen in hypoxia, as TRAP1 is anyway down regulated by prolonged hypoxic exposure (unpublished observation). In agreement with our results, it has been recently demonstrated that TRAP1 knockdown in esophageal squamous cell carcinoma cell lines supressed cell proliferation and survival [7]. Besides its role on proliferation, TRAP1 knockdown has also associated with altered mitochondrial functions such as ATP production and mitochondrial membrane potential [6].

Data from microarray mRNA expression analysis [8] further supported the conclusion that TRAP1 has an effect on cell cycle, either in mitochondria or in the nucleus, since in TRAP1 positive cells there are higher levels of genes promoting cell proliferation through the TNK pathway, as compared to TRAP1 negative cells. These TRAP1 negative cells showed higher levels of genes controlling cell motility and metastatic spread. A last striking result was that no differences were detected as far as levels of expression of apoptosis and metabolism regulating genes is concerned. These data fit well with the observations that both the mitochondrial and nuclear chaperoning activity of TRAP1 is triggered by a shock and it is performed by the already present protein which control post translational changes resulting into apoptosis. TRAP1 exert on these pathways by acting as a chaperone as part of unfolded protein response following a shock.

But what is eventually the clinical meaning as far as gain or loss of expression and its intra cellular localization is concerned? Very limited so far the number of studies looking at the value of TRAP1 as predictive and prognostic marker on series of patients. Although none of the studies has a large enough population to reach firm conclusions, some indications are emerging: TRAP1 cytoplasmic expression appears to be higher in metastases compared to primary tumours and overall survival of patients with positive tumours is consistently shorter across different studies [6]. In two of our papers we also looked at nuclear expression, that we found associated with RB1 presence and, in breast cancer, a better outcome[5]. However we have to recognise that data so far are insufficient to have a proper idea of the prognostic role of TRAP1.

Therefore TRAP1 seems to have two opposite functions as far as cell cycle regulation is concerned: in normoxia, it promotes cell cycle likely through the TNF pathway and protects from apoptosis; however, following a hypoxic shock it moves to the nucleus where it is essential for RB1 to remain in an active conformation to induce, within 24 hours, a rapid, short-term slowing down of proliferation. If hypoxia persists, TRAP1 cytoplasmic levels will then progressively decrease (unpublished results) and after 24- 48 hours the proliferation rate the proliferating fraction will also decrease due to switching off of the pathway to whom TRAP1 belongs.

As new roles play by TRAP1 within the cell keeps appearing, many aspect of its biology still remain to be unveiled.
